# Same Pathogen, Different Manifestations: A Case of Extrapulmonary Tuberculosis

**DOI:** 10.7759/cureus.50436

**Published:** 2023-12-13

**Authors:** Joana A Cabrera, Margarida Mota

**Affiliations:** 1 Internal Medicine, Centro Hospitalar de Vila Nova de Gaia/Espinho, Vila Nova de Gaia, PRT; 2 Infectious Diseases, Centro Hospitalar de Vila Nova de Gaia/Espinho, Vila Nova de Gaia, PRT

**Keywords:** mycobacterium tuberculosis, anti-tuberculosis therapy, tuberculous meningitis, hepatic tuberculosis, extrapulmonary tuberculosis

## Abstract

Tuberculosis (TB) remains the most prevalent contagious disease worldwide and a significant cause of morbidity, ranking as the second most deadly disease globally. The transmission of the disease occurs through aerosols via the respiratory route, predominantly affecting pulmonary tissue. However, the pathogen can disseminate and infect any organ within the body. Up to 15% of patients exhibit extrapulmonary involvement.

The case involves a 59-year-old male who presented to the emergency department complaining of abdominal pain and subfebrile episodes, without any other significant symptoms or findings on physical examination. Laboratory investigations revealed elevated inflammatory markers and abnormal liver biochemistry parameters. A computed tomography (CT) scan showed a neoformative lesion in the liver - a collection with a vascularized, thick, irregular wall. This raised the possibility of a potentially hypervascular hepatic neoformation or an encysted inflammatory lesion. The patient was started on empirical broad-spectrum antibiotics and was admitted to the Internal Medicine ward for further investigation. Later, the patient began to exhibit a decline in overall condition, a slowed and less complex speech pattern, loss of balance, and distal tremors in the upper limbs, as well as a symmetric and distal reduction in strength in all four limbs. A cerebral CT scan revealed no significant abnormalities, and a lumbar puncture yielded no immediate notable findings. Simultaneously, a repeated abdominal CT scan showed the previously known hepatic lesion, albeit with features more indicative of a multiloculated collection. An aspirative biopsy of the hepatic abscess was conducted. From the extensive analysis conducted, a positive PCR result for mycobacterium tuberculosis was identified in both the pus from the hepatic abscess and the cerebrospinal fluid. This led to the conclusion that the case presented was an instance of extrapulmonary TB involving the liver and the central nervous system. Following the identification of the causative agent, the patient commenced antibacterial therapy comprising rifampicin, ethambutol, and isoniazid with adjunctive dexamethasone. Despite targeted treatment and instituted supportive therapy, the patient exhibited an unfavorable progression and eventually succumbed 57 days after diagnosis.

This case highlights an unusual manifestation of a patient with disseminated extrapulmonary TB, emphasizing the importance of early diagnostic suspicion for clinicians. The unfavorable disease progression despite appropriate targeted treatment prompts reflection on whether the delay in diagnosis and provision of anti-TB drugs may have played a major role in the prognosis of the patient.

## Introduction

Tuberculosis (TB), caused by the *Mycobacterium tuberculosis complex bacterium*, stands as one of the oldest known diseases in humans. Despite the widespread use of the *Mycobacterium bovis* BCG vaccine, it remains a significant cause of morbidity worldwide, ranking as the second most deadly disease globally, following human immunodeficiency virus (HIV) infection (in the AIDS stage). Despite being classified as a country with low incidence, in Portugal, in 2018, the notification rate was 16.6 cases per 100,000 inhabitants [1,2].

Infection with *Mycobacterium tuberculosis* occurs through aerosols via the respiratory route, making it currently the most prevalent contagious disease worldwide. Following respiratory inoculation, the pathogen can disseminate and infect any organ within the body [1,2]. TB predominantly affects pulmonary tissue; however, up to 15% of patients exhibit extrapulmonary involvement. Extrapulmonary TB may result from hematogenous dissemination originating from an active primary focus in the lungs to other organic system(s) within the body, and this manifestation can occur years after the initial pulmonary infection. Dissemination may also occur through surgical or diagnostic manipulation of a previously infected organ. A normal chest radiograph or negative laboratory tests do not exclude extrapulmonary TB, a diagnosis that necessitates a high index of diagnostic suspicion [3]. Most cases of extrapulmonary TB occur in organs that do not offer optimal conditions for bacillary growth, leading to an insidious onset of the disease [4].

The most common extrapulmonary forms of TB include pleural, lymphatic, osteoarticular, genitourinary, and intestinal, although virtually any organ within the body can be affected. Tuberculous pleurisy stands as the most prevalent extrapulmonary form of TB in immunocompetent adults [3].

## Case presentation

The case involves a 59-year-old male individual dating back to the year 2019. Pertinent medical history includes hospitalization due to idiopathic acalculous cholangitis in July 2019, during which he underwent empirical antibiotic therapy with piperacillin/tazobactam, exhibiting a favorable clinical and laboratory response. Following discharge, the patient returned home, attended follow-up outpatient consultations, and remained asymptomatic until the subsequent episode reported thereafter. No other significant personal medical history was identified, and the patient did not adhere to any regular medication. The patient had an up-to-date Portuguese national vaccination plan, which, however, did not include BCG vaccination during his childhood. There were no recent travels abroad from Portugal, close contact with migrants, or apparent contact with individuals exhibiting illness.

Three months prior to index admission, the patient presented to the emergency department complaining of abdominal pain and subfebrile episodes. No other significant symptoms were identified during the medical history. Physical examination did not reveal any noteworthy changes in the patient's general condition. Upon admission to the emergency department, laboratory investigations showed elevated inflammatory markers and abnormal liver biochemistry parameters (Table 1).

**Table 1:** Relevant analytical values upon admission to the emergency department From the analytical study conducted upon admission to the emergency department, here are some of the analytical values showing deviations from the normal range or deemed relevant for the present case. The patient's test value is provided alongside the institutional normal value limit.

Parameters	Patient Value	Reference Range
Hemoglobin	13,2 g/dL	N: 13.8 - 17.2 g/dL
Leukocytes	7.120/uL	N: 4,500 - 11,000/µL
Neutrophils	5.150/uL	N: 1,800 - 7,700/µL
Oxaloacetic transaminase	60 u/L	N: 5 - 40 U/L
Pyruvic transaminase	46 u/L	N: 7 - 56 U/L
Lactate dehydrogenase	144 u/L	N: 140 - 280 U/L
Total bilirubin	1,7 mg/dL	N: 0.3 - 1.2 mg/dL
Direct bilirubin	1,48 mg/dL	N: 0.1 - 0.3 mg/dL
C-reactive protein	8,48 mg/dL	N: < 1.0 mg/dL
HIV Antibody	Negative	-
Hepatitis C Antibody	Negative	-
Hepatitis B Antibody	Negative	-

**Figure 1:**
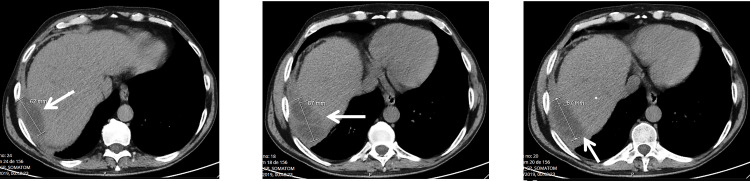
Abdominal CT scan performed upon admission to the emergency department With an arrow, the newly identified hepatic lesion is indicated, and its dimensions are marked in millimeters.

The abdominal ultrasound was inconclusive, prompting an abdominal computed tomography (CT) scan. The CT scan revealed a suspected neoformative lesion adjacent to the posterior aspect of the right hepatic lobe, where a collection with a vascularized, thick, irregular wall was visualized. There wasn't any extra ou intra-hepatic biliary duct dilation. This structure appeared elongated, septated, and measured 10 x 7.5 x 5 cm. The identified image raised the possibility of a potentially hypervascular hepatic neoformation, characterized as an older, encapsulated, septated subcapsular hematoma, or an encysted inflammatory lesion (Figure 1).

Following this emergency episode, the patient was admitted for surveillance and further diagnostic investigation. Due to suspected infectious processes, empirical antibiotic therapy with meropenem was initiated subsequent to blood culture sampling (with negative results).

The hospitalization progressed without major complications until 13 days after admission, when the patient began to exhibit a decline in overall condition. There was a progressively slowed and less complex speech pattern, loss of balance, and distal tremors in the upper limbs, as well as a symmetric and distal reduction in strength in all four limbs. No other deficits were observed in the neurological examination or any new alterations in the general physical examination.

**Figure 2:**
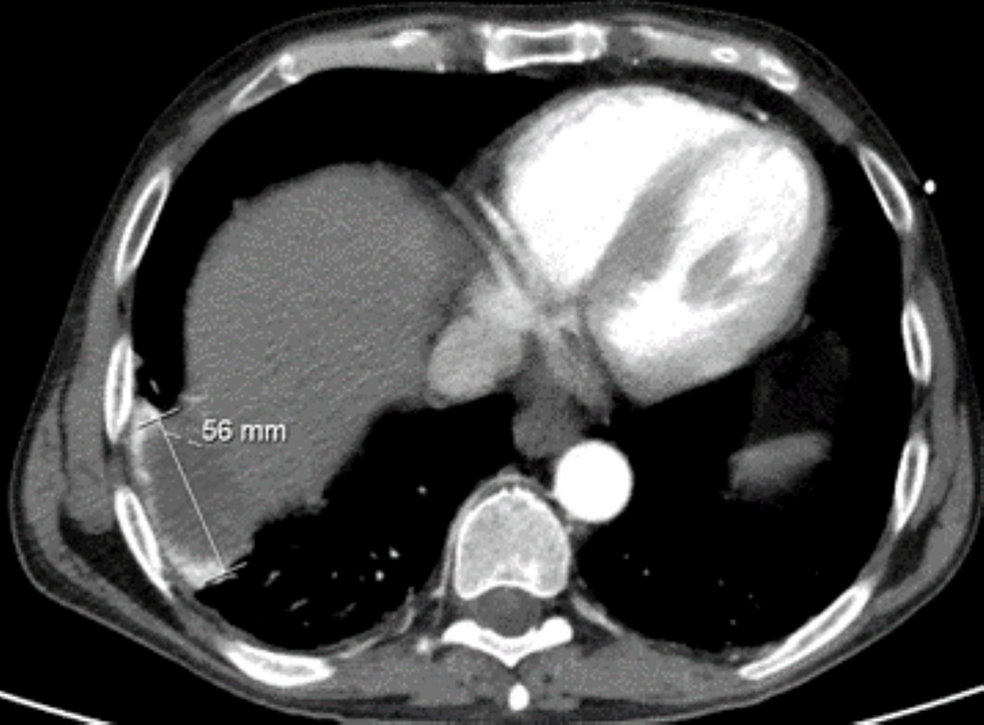
Abdominal CT scan for reassessment following clinical deterioration The dimensions of the hepatic lesion are marked on the figure in millimeters.

In this context, a revaluation abdominal CT scan was performed, revealing the previously known hepatic lesion, albeit with features more indicative of a multiloculated collection and slightly smaller dimensions compared to the prior examination (Figure 2). An aspirative biopsy of the hepatic abscess was conducted, and specimens were collected for broad-spectrum microbiological screening.

Simultaneously, due to the neurological symptoms, the patient underwent cranial computed tomography imaging, which revealed no significant abnormalities, and a lumbar puncture, yielding no notable findings in the immediate cytological examination. All microbiological samples were processed for bacterial and mycobacterial culture, alongside various polymerase chain reaction (PCR) analyses targeting agents with high diagnostic suspicion.

From the extensive analysis conducted, a positive PCR result for *Mycobacterium tuberculosis* was identified in both the pus from the hepatic abscess and the cerebrospinal fluid, leading to the conclusion that the case presented was an instance of extrapulmonary TB involving the liver and the central nervous system (CNS). Subsequently, a bronchoscopy with bronchoalveolar lavage analysis was performed, also identifying *Mycobacterium tuberculosis* by PCR, but no pulmonary lesions were identified. Cultural tests using culture media for mycobacterial growth were obtained but did not yield conclusive results. Therefore, the diagnosis was exclusively established through pathogen identification via PCR. Drug resistance/sensitivity tests were not conducted.

Following the identification of the causative agent, the patient commenced antibacterial therapy comprising rifampicin, ethambutol, and isoniazid, with adjunctive dexamethasone. Although the antibacterial regimen did not initially include pyrazinamide (due to concerns regarding toxicity in a patient with underlying hepatic alterations), there was a deterioration in cholestatic enzyme levels. Consequently, isoniazid was temporarily suspended for seven days, leading to the normalization of liver biochemistry parameters.

Despite targeted treatment and instituted supportive therapy, the patient exhibited an unfavorable progression and eventually succumbed 57 days after diagnosis. The patient passed away due to multiorgan failure in the context of septic shock with minimal response to antimicrobial agents. No subsequent nosocomial infections or other significant occurrences were identified.

## Discussion

Extrapulmonary TB presents intra-abdominal manifestations in 11% of patients, with the most common site being the ileocecal region. This manifestation can occur as part of active pulmonary disease or as a primary infection without pulmonary involvement, as observed in the described case. It can involve any segment of the gastrointestinal tract, associated viscera, and peritoneum. Hepatic, splenic, or pancreatic TB forms are rare and typically associated with disseminated miliary TB, more common in immunocompromised patients. Hepatic TB may manifest as a pseudotumor, cholangitis, hepatitis, hepatic abscess (localized liver disease), or widespread micronodular involvement (miliary hepatic TB - present in 80% of disseminated TB cases). Local hepatic TB occurs in about 20% of hepatic TB cases, where the microorganism reaches the liver from the intestinal tract via the portal vein or gastrointestinal lymphatics and may represent a reactivation of latent TB infection. This form of TB generally responds well to treatment, usually without sequelae [2,4,5].

The diagnosis of gastrointestinal TB is often delayed due to its nonspecific clinical presentation. Suspecting abdominal TB should be considered in patients with relevant clinical manifestations in regions with a significant epidemiological context. Diagnosis of gastrointestinal TB is frequently delayed owing to its nonspecific clinical presentation. While some patients may be asymptomatic, common symptoms of gastrointestinal TB include fever, anorexia, weight loss, nausea, vomiting, abdominal pain, diarrhea, and constipation. During physical examination, signs such as pallor, abdominal distension, ascites, hepatomegaly, splenomegaly, rectal bleeding, and abdominal mass may be detected, depending on the involved area of the gastrointestinal tract [2,4,5].

Early initiation of anti-tubercular therapy is crucial in reducing morbidity and mortality. Standard anti-TB therapy is typically adequate for gastrointestinal TB. When left untreated, it carries a mortality rate ranging from 6% to 20%. Surgery is not mandatory unless complications arise that do not respond to medical therapy [3,4].

TB of the CNS stands among the less common yet most devastating forms of mycobacterial infection in humans. CNS TB encompasses three clinical categories: tuberculous meningitis, intracranial tuberculoma, and tuberculous spinal arachnoiditis-predominantly found in regions with a high incidence of TB. Tuberculous meningitis, often occurring independently of infection in other extrapulmonary sites, accounts for 1% of all TB cases and 5% of all extrapulmonary TB cases [2,6,7].

The early diagnosis of tuberculous meningitis can be challenging but holds paramount importance as it profoundly influences clinical outcomes, depending on the stage at which therapy is initiated. A high index of clinical suspicion is crucial in diagnosing this manifestation of TB. Typically, a patient with tuberculous meningitis presents with a subacute and progressive febrile illness that progresses through three discernible phases. The disease commences with prodromal symptoms of malaise, fatigue, low-grade fever, intermittent headaches, occasional nonspecific discomfort in the neck or back, and subtle personality changes. Within 2 to 3 weeks, a more distinct meningitic phase ensues as the patient experiences prolonged headache, meningismus, vomiting, mild confusion, and various degrees of cranial nerve and long tract signs. During this phase, the disease's pace may rapidly accelerate into the paralytic phase: delirium followed by stupor and coma, seizures, and multiple focal neurological deficits. In untreated cases, death typically occurs 5 to 8 weeks after the onset of the disease [6-8].

The anti-TB therapy for CNS TB should be initiated promptly upon clinical suspicion and should not be delayed until laboratory confirmation, as this timing significantly impacts clinical outcomes. The treatment for this form of extrapulmonary TB relies on a combination of anti-TB drugs similar to pulmonary disease, with ongoing discussions about the potential use of higher-than-standard doses [8].

## Conclusions

TB remains the disease with the highest number of documented cases of contagion worldwide. Although less prevalent, approximately 1 in 5 patients will present with forms of extrapulmonary TB, posing diagnostic challenges and increased morbidity and mortality.

The clinical manifestations of extrapulmonary TB are diverse and vary depending on the affected system, often presenting with atypical symptoms, requiring a high degree of suspicion. Early diagnostic consideration is crucial, and anti-TB therapy should be initiated upon clinical suspicion without delay for laboratory confirmation, as this delay significantly influences clinical outcomes. When feasible, conducting tests for microbial resistance and susceptibility should be pursued to guide therapy, although this remains a viable option within a very limited number of reference centers in a timely manner. Treatment for extrapulmonary TB is based on a combination of anti-TB drugs similar to pulmonary disease. The concomitant use of steroids has been studied and recommended, especially in severe cases with apparently less response to therapy, particularly concerning involvement of the central nervous system.

The case in question highlights an unusual manifestation of a patient with disseminated extrapulmonary TB, emphasizing the importance of early diagnostic suspicion for clinicians and raising awareness about the side effects of anti-tubercular therapy. The unfavorable disease progression despite appropriate targeted treatment prompts reflection on whether the delay in diagnosis and provision of anti-TB drugs may have played a major role in the prognosis of the patient in question.
